# Results of definitive radiotherapy with concurrent chemotherapy for maxillary sinus carcinomas with neck lymph node metastasis

**DOI:** 10.1093/jrr/rraa120

**Published:** 2020-12-07

**Authors:** Takanori Abe, Satoshi Saito, Misaki Iino, Tomomi Aoshika, Yasuhiro Ryuno, Tomohiro Ohta, Mitsunobu Igari, Ryuta Hirai, Yu Kumazaki, Yasuhiro Ebihara, Mitsuhiko Nakahira, Masashi Sugasawa, Shin-ei Noda, Shingo Kato

**Affiliations:** Departments of Radiation Oncology; Departments of Radiation Oncology; Departments of Radiation Oncology; Departments of Radiation Oncology; Departments of Radiation Oncology; Departments of Radiation Oncology; Departments of Radiation Oncology; Departments of Radiation Oncology; Departments of Radiation Oncology; Head and Neck Surgery, International Medical Center, Saitama Medical University, Hidaka, Japan; Head and Neck Surgery, International Medical Center, Saitama Medical University, Hidaka, Japan; Head and Neck Surgery, International Medical Center, Saitama Medical University, Hidaka, Japan; Departments of Radiation Oncology; Departments of Radiation Oncology

**Keywords:** maxillary sinus carcinoma, neck lymph node metastasis, radiotherapy, concurrent chemotherapy, selective arterial chemo-infusion

## Abstract

The purpose of this study was to describe the results of definitive radiotherapy (RT) with concurrent chemotherapy for maxillary sinus carcinomas (MSCs) with neck lymph node metastasis to clarify its limitation. Local control (LC), progression-free survival (PFS) and overall survival (OS) rates were calculated using the Kaplan–Meier method and were compared between subgroups using the log rank test. Toxicity was classified using common terminology criteria of adverse events version 5.0. Eighteen patients with inoperable MSC with neck lymph node metastasis including 12 men and 6 women with a median age of 67 years were analyzed. The histologic diagnoses were as follows: 16 patients had squamous cell carcinomas and 2 had other histology. Four patients had stage T3 MSC, 6 had T4a and 8 had T4b. Among 18 patients, 7 received concurrent systemic chemotherapy and 11 received selective arterial chemo-infusion. The median follow-up period was 17 months. The 2-year LC, PFS and OS rates for the entire cohort were 34, 31 and 46%, respectively. No significant differences were observed for LC, PFS and OS rates between systemic chemotherapy and selective arterial chemo-infusion cohorts. Grade 3 or higher acute toxicity, including both non-hematological and hematological, was observed in nine patients (50%), while no grade 3 or higher late toxicity was observed. In conclusion, we described the results of definitive RT for MSCs with neck lymph node metastasis. Local recurrence of primary tumor was a frequent pattern of failure and it should be addressed in future study.

## INTRODUCTION

Maxillary sinus carcinomas (MSCs) account for ~1–4% of all head and neck cancers [1, 2]. Multidisciplinary approaches including surgery, radiotherapy (RT) and chemotherapy are established treatments for locally advanced MSCs [3]. However, surgical resection for advanced disease can sometimes be problematic because of extensive infiltration of the tumor or for other medical reasons. For such patients, definitive RT with concurrent chemotherapy has been reported to be effective and feasible [4–10]. However, results of definitive RT with concurrent chemotherapy were mainly obtained in patients without neck lymph node metastasis because of the low incidence of lymph node metastasis, even in advanced MSCs [4–13]. The utility of definitive RT with concurrent chemotherapy for MSC with neck lymph node metastasis has yet to be determined. Therefore, in this study, we collected data from MSC patients with neck lymph node metastasis who were treated with definitive RT with concurrent chemotherapy and analyzed the efficacy, pattern of failure and treatment toxicity.

## MATERIALS AND METHODS

### Patients

Patients with MSCs with neck lymph node metastasis who received definitive RT with concurrent chemotherapy between 2008 and 2019 at our hospital were retrospectively analyzed. All primary tumors were diagnosed histologically. Clinical stage was determined using contrast-enhanced computed tomography (CT), fluorodeoxyglucose-positron emission tomography (FDG-PET)/CT and gadolinium-enhanced magnetic resonance imaging (MRI), and classified according to the Union for International Cancer Control 8th edition classification of malignant tumors. Diagnosis of lymph node metastasis was made by cytology or clinical findings as follows: (i) short axis of swollen lymph node was >1 cm and (ii) abnormal uptake of FDG on PET/CT image. This study was approved by the Institutional Review Board and was carried out in an accordance with the Declaration of Helsinki.

### Treatment

RT was performed using 6-MV X-ray beams with CT image simulation. A CT image for treatment planning was acquired using a thermos plastic shell. The thickness of the CT image was 1.25 mm. The treatment method used was conventional 3D conformal RT (3D-CRT) in the early period, and subsequently, all patients were treated with intensity-modulated RT (IMRT). Basic planning methods are shown below, but some patients were treated with a different method by a former physician at our hospital. Gross tumor volume (GTV) was defined in treatment planning using CT images with reference to MRI. The clinical target volume (CTV) consisted of a 5–10 mm margin in all directions from the GTV to encompass microscopic disease extensions (CTV-primary) and prophylactic lymph node areas adjacent to metastatic lymph nodes (CTV-LN). The planning target volume (PTV) was created from the CTV-primary and CTV-LN with a 3 mm margin to compensate for setup error (PTV-primary and PTV-LN). The prescribed dose was basically 70 Gy in 35 fractions. Nowadays, we treat all the patients with 70 Gy in 35 fractions. Some patients in the early period whose treatment was performed by a former doctor of our hospital were treated with a different dose. The aim was to cover the PTV-primary with 95% of the prescribed dose and to cover the PTV-LN with 85–90% of the prescribed dose. Systemic chemotherapy or arterial chemo-infusion was determined by the clinician. The chemotherapy regimen was tri-weekly systemic administration of cisplatin at 100 mg per square meter of body surface area or weekly selective arterial chemo-infusion of cisplatin at 100 mg per square meter of body surface area during RT.

### Evaluation

Local recurrence included recurrence of the primary tumor and recurrence in the irradiated neck lymph node. Local control (LC) was defined as being free from local recurrence. Progression-free survival (PFS) was calculated as the interval between the initiation of treatment and local recurrence or distant metastasis. Overall survival (OS) was calculated as the interval between the initiation of treatment and the last follow-up or death. All acute and late toxicities were evaluated using the National Cancer Institute Common Terminology Criteria for Adverse Events version 5.0. Dose–volume parameters such as minimum dose in the most irradiated 90, 95 and 98% of GTV (GTV D90, GTV D95 and GTV D98) were analyzed for their correlation with LC.

### Statistical analyses

LC, PFS and OS rates were calculated using the Kaplan–Meier method and were compared between subgroups using the log rank test. To determine the optimal cut-off value of the dose–volume parameter to predict LC, receiver operating characteristic analysis was performed. Mean parameters in two groups were compared using the Student’s *t*-test. Differences in categorical variables between two groups were compared using the Chi-square test. *P* < 0.05 was considered statistically significant. All statistical analyses were performed using IBM SPSS Statistics for Windows, version 25.0 (SPSS Inc, Armonk, NY, USA).

## RESULTS

### Patient and treatment characteristics

**Fig. 1.**
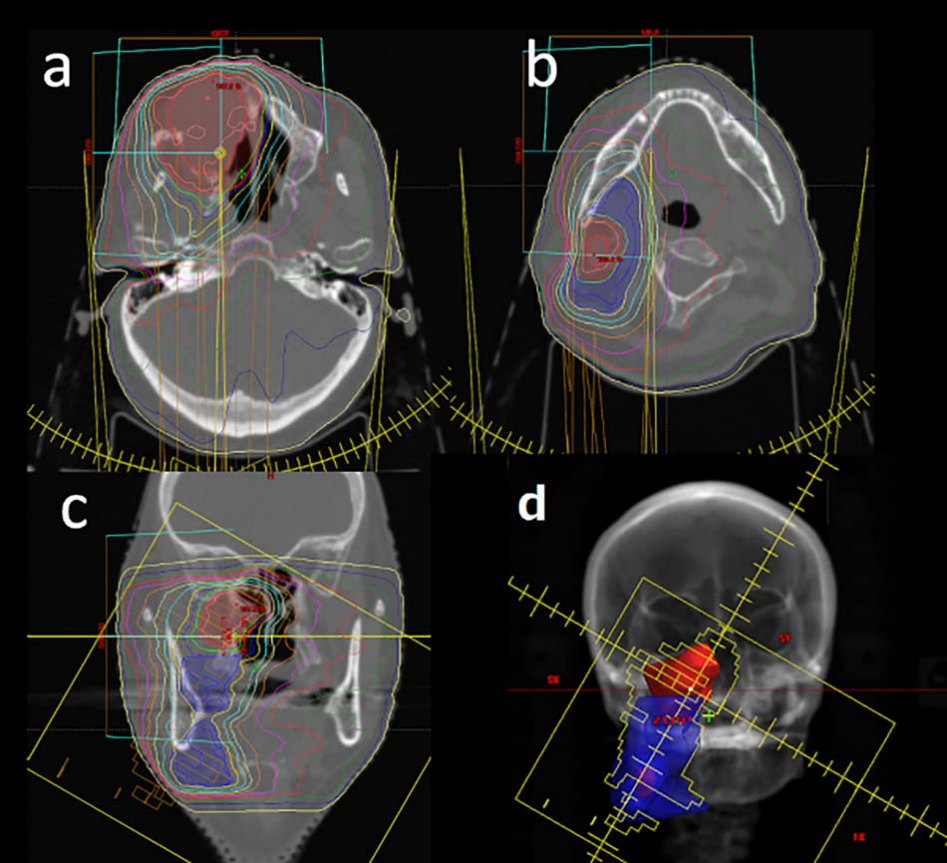
A representative case treated with intensity modulated radiotherapy is shown. (**a**) Axial CT image at the level of primary tumor. (**b**) Axial CT image at the level of lymph node metastasis. (**c**) Coronal CT image. (**d**) Beam’s eye view of the treatment plan. The primary tumor is shown in red mesh and the neck lymph node area is shown in blue mesh. Blue line, 95% isodose line of prescribed dose.

Between January 2008 and July 2019, 18 patients with MSCs with neck lymph node metastasis were treated. Representative cases treated with IMRT are shown in Fig. 1. Their median age was 67 years (range: 46–83), and there were 12 male and 6 female patients. Histologic diagnoses were as follows: 16 patients with squamous cell carcinomas, 1 with a poorly differentiated carcinoma and 1 with a lymphoepithelial carcinoma. There were 4 patients with stage T3 disease, 6 with T4a and 8 with T4b. There were 7 patients with N1 disease, 7 with N2b, 3 with N2c and 1 with N3b. Diagnosis of lymph node metastasis was made by cytology in 2 patients and the other patients were clinically diagnosed by CT, MRI image and abnormal uptake of FDG on PET/CT image. The median prescribed dose to treat the GTV was 66 Gy (range: 46–70). Among the 18 patients, 7 were treated with concurrent systemic chemotherapy and 11 were treated with selective arterial chemo-infusion. The artery for chemo-infusion was carefully selected under CT angiography. Maxillary artery, facial artery, transverse facial artery, middle meningeal artery, ascending pharyngeal artery and distal part of external carotid artery were used according to the vascular supply of the tumor. Regarding RT method, 7 patients were treated with 3D-CRT and 11 patients were treated with IMRT. These characteristics are summarized in Table 1.

**Table 1:** Patient and tumor characteristics (*n* = 18)

Characteristic		Systemic chemotherapy (*n* = 7)	Arterial chemo-infusion (*n* = 11)	*P* value
Age, years, median (range)		61 (48–73)	68 (46–81)	0.790
Sex	Male	5 (71)	7 (64)	0.732
	Female	2 (29)	4 (36)	
Histopathological type, *n* (%)	Squamous cell carcinoma	5 (71)	11 (100)	0.06
	Other	2 (29)	0 (0)	
T classification, *n* (%)	T3	1 (14)	3 (27)	0.232
	T4a	4 (57)	2 (18)	
	T4b	2 (29)	6 (55)	
N classification, *n* (%)	N1	3 (43)	4 (36)	0.224
	N2b	1 (14)	6 (55)	
	N2c	2 (29)	1 (9)	
	N3b	1 (14)	0 (0)	
Clinical stage, *n* (%)	III	0 (0)	3 (27)	0.137
	IVA	4 (57)	2 (18)	
	IVB	3 (43)	6 (55)	
Gross tumor volume, cm^3^*		115 (39–299)	79 (39–205)	0.289
Radiation method, *n* (%)	IMRT	2 (29)	6 (55)	0.28
	3D-CRT	5 (71)	5 (45)	
Total radiation dose	<60 Gy	1 (14)	2 (18)	0.792
	60–66 Gy	3 (43)	3 (27)	
	70 Gy	3 (43)	6 (55)	

^*^median value (range) were shown.

### Treatment efficacy

**Fig. 2.**
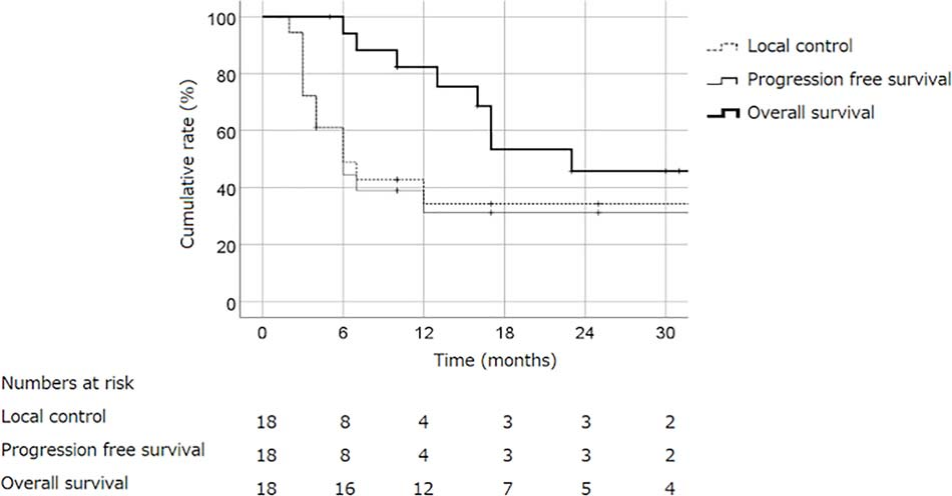
The cumulative LC, PFS and OS rates of the entire cohort (*n* = 18) are shown. The 2-year LC, PFS and OS rates were 34, 31 and 46%, respectively.

**Fig. 3.**
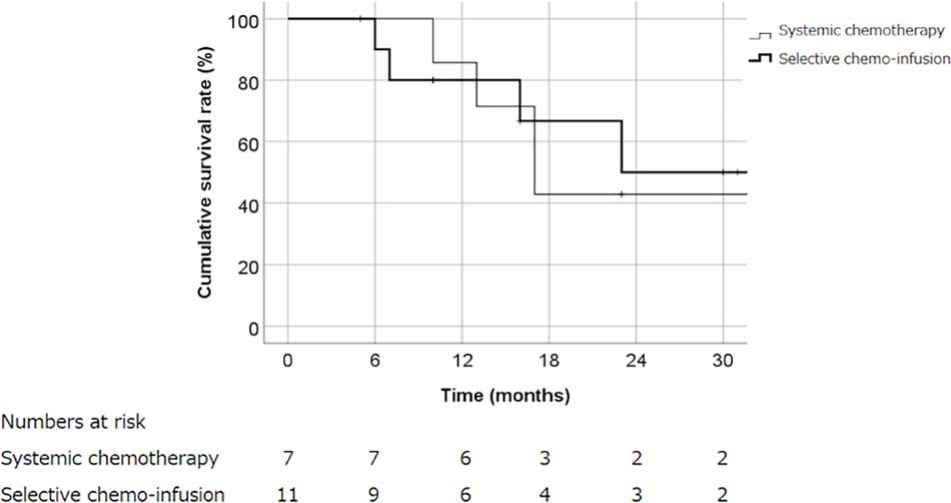
Cumulative OS rate of patients treated with systemic chemotherapy or selective arterial chemo-infusion are shown. OS rates were 43% in patients treated with systemic chemotherapy, while rates in patients treated with selective arterial chemo-infusion were 50% (*P* = 0.72).

With a median follow-up of 17 months (range: 6–138), the 2-year LC, PFS and OS rates for all patients were 34, 31 and 46%, respectively (Fig. 2). The 2-year LC, PFS and OS rates were 29, 29 and 43%, respectively, in patients treated with concurrent systemic chemotherapy and RT, while rates in patients treated with selective arterial chemo-infusion were 35, 30 and 50%, respectively. Significant differences were not observed for LC, PFS and OS rates between systemic chemotherapy and selective arterial chemo-infusion treatments (Fig. 3). Factors such as GTV, T classification, N classification, dose of RT and method of RT were also analyzed using the log rank test for their correlation with LC, but no correlation was found. Grade 3 acute toxicity including both non-hematological and hematological occurred in 9 of 18 patients, and all adverse events improved. One patients developed grade 4 sepsis during treatment. This patients showed high fever, low blood pressure and tachycarcdia, which indicate septic shock, and was treated with continuous drip infusion of vasopressor and antibiotics. A blood culture test was positive for Klebsiella pneumonia, which indicates infection from the primary tumor. The patient’s condition was improved by the above-mentioned treatment and RT could be resumed. The incidence of grade 3 or higher acute toxicity was not significantly different between systemic chemotherapy and arterial chemo-infusion treatments (*P* = 0.147). One patient developed grade 2 dysgeusia and one developed grade 2 dysphagia as late toxicities, which are summarized in Table 2.

**Table 2:** Incidence of late toxicity (*n* = 18)

Type of toxicity	Number of patients (%)				
	Grade 0	Grade 1	Grade 2	Grade 3	Grade 4–5
Epistaxis	17 (94)	1 (6)	0 (0)	0 (0)	0 (0)
Nasal congestion	15 (83)	3 (17)	0 (0)	0 (0)	0 (0)
Dysphagia	17 (94)	0 (0)	1 (6)	0 (0)	0 (0)
Dysgeusia	17 (94)	0 (0)	1 (6)	0 (0)	0 (0)
Watering eyes	17 (94)	1 (6)	0 (0)	0 (0)	0 (0)
Postnasal drip	17 (94)	1 (6)	0 (0)	0 (0)	0 (0)
Hearing impairment	17 (94)	1 (6)	0 (0)	0 (0)	0 (0)
Osteonecrosis of the jaw	17 (94)	1 (6)	0 (0)	0 (0)	0 (0)

None of the dose–volume parameters including GTV D90, GTV D95 and GTV D98 showed significant differences regarding LC. However, with GTV D95, the 1-year LC rate was 63% in the groups above the cut-off value (GTV D95; 71.8 Gy), while in the groups below the cut-off value, the 1-year LC rate was 25%, although there was no significant difference between cut-off groups (*P* = 0.229). Mean GTV D95 was 54.9 Gy in the 3D-CRT group, while that in the IMRT group was 72.5 Gy; this difference was statistically significant (*P* = 0.00).

### Pattern of failure

A total of 12 patients developed local recurrence during the follow-up period. The clinical course of these patients is summarized in Table 3. Among the 12 patients with local recurrence, 9 patients relapsed only in the primary tumor, 2 in the neck lymph node and 1 in both the primary tumor and the neck lymph node. Four patients received salvage surgery for local recurrence. Among the 4 patients who received salvage surgery, 3 patients were successfully salvaged and 1 died of re-recurrence. Re-irradiation or chemotherapy was not performed for the patients with recurrence who were not suitable for salvage surgery due to their poor general condition. Distant metastasis was observed in 3 patients, and the sites of distant metastasis were lung, liver and bone. At the last follow-up, 9 patients had died. Among the 9 patients who died, 6 died of recurrence in the primary tumor, 2 died of recurrence in neck lymph node and 1 died of a distant metastasis.

**Table 3:** Clinical course of 12 patients with local recurrence

Case	Prescribed dose (Gy)	Chemotherapy method	Site of recurrence	Time to recurrence (months)	Treatment for recurrence	Results	Time from recurrence to death/last follow-up date (months)
1	46	Systemic chemotherapy	Primary tumor	3	Best supportive care	Died from primary disease	13
2	60	Arterial chemo-infusion	Primary tumor and multiple lymph node	3	Best supportive care	Died from primary disease	3
3	60	Arterial chemo-infusion	Primary tumor	6	Best supportive care	Died from primary disease	10
4	52	Arterial chemo-infusion	Multiple lymph node	3	Best supportive care	Died from primary disease	20
5	70	Arterial chemo-infusion	Primary tumor	3	Salvage surgery	Alive without evidence of disease	38
6	70	Arterial chemo-infusion	Primary tumor	12	Salvage surgery	Alive without evidence of disease	14
7	60	Systemic chemotherapy	Primary tumor	4	Best supportive care	Died from primary disease	9
8	70	Systemic chemotherapy	Primary tumor	4	Best supportive care	Died from primary disease	13
9	70	Arterial chemo-infusion	Primary tumor	7	Salvage surgery	Died from recurrence after surgery	7
10	64	Systemic chemotherapy	Primary tumor	6	Best supportive care	Died from primary disease	28
11	60	Arterial chemo-infusion	Primary tumor	86	Salvage surgery	Alive without evidence of disease	31
12	70	Systemic chemotherapy	Multiple lymph node	2	Best supportive care	Died from primary disease	8

## DISCUSSION

In this study, the 2-year OS rate was 46%. There are no reports of treatment results for cases limited only to MSC with positive lymph node metastasis. Shiga *et al*. reported that the 2-year OS rate was ~67% after combined selective arterial chemo-infusion and RT treatment for MSCs [7]. In their report, the proportion of patients with neck lymph node metastasis was 20%, while all the patients in the current study had lymph node metastasis. Nishimura *et al*. reported that the 2-year OS rate was ~60% after concurrent chemoradiation therapy for MSCs without neck lymph node metastasis [6]. We consider that the 2-year OS rate in the current study was reasonable if we take the difference in lymph node metastasis rate into account. We think that definitive RT with concurrent chemotherapy should be considered even for patients with unresectable MSCs with neck lymph node metastasis, although outcomes will need to be improved.

The reported 2-year LC rates of definitive RT for MSCs without neck lymph node metastasis ranged from 55 to 70% [4, 8, 14]. The 2-year LC rate in this study was 34%, which was lower than those in these other reports. Generally, MSCs with neck lymph node metastasis are more advanced diseases with wide infiltration into surrounding organs compared with MSCs without neck lymph node metastasis, and that may be why our result was different from the reported outcomes for MSCs without neck lymph node metastasis. Actually, Ono *et al*. reported that a positive lymph node was a poor prognostic factor for PFS in the treatment of MSCs with selective arterial chemo-infusion and RT [14]. Factors such as GTV, T classification, N classification, dose of RT and method of RT were also analyzed using the log rank test for their correlation with LC, but no correlation was found. To find a favorable prognostic factor, we also analyzed the relationship between dose–volume parameters and LC but could not find any significant parameter. However, with GTV D95, the 1-year LC rate was 63% in the groups whose GTV D95 was >102.5%, while in the groups whose GTV D95 was below the cut-off value, the 1-year LC rate was 25%, although there was no significant difference (*P* = 0.229). The mean GTV D95 was 93.7% in the 3D-CRT group, while that in IMRT group was 103.6%, and this difference was statistically significant (*P* = 0.00). Longer follow-up with a larger number of patients will be necessary to clarify whether sufficient dose coverage of the tumor with IMRT could achieve a better LC rate or not.

For the treatment of MSCs with neck lymph node metastasis, systemic chemotherapy has an effect on both the primary tumor and the neck lymph node and possible distant metastasis, while arterial chemo-infusion has an effect only on the primary tumor.

It is difficult to determine which therapy has a better outcome for MSCs with neck lymph node metastasis. Regarding the cause of death in this study, among 9 patients who died of their MSC, 6 patients died of recurrence of their primary tumor. This might suggest that LC of the primary tumor is essential to sustain survival in MSC patients with neck lymph node metastasis. However, in this study, the 2-year LC and OS rates were not significantly different between patients treated with systemic chemotherapy and those treated with selective arterial chemo-infusion. It might be because in this study the number of patients was too small to detect a difference between treatment methods. Further study will be necessary to determine whether systemic chemotherapy or selective arterial chemo-infusion contributes to longer survival in MSC patients with neck lymph node metastasis.

The incidence of grade 3 or higher acute toxicities was 50% in this study, although the most frequent toxicities were acute mucositis and dermatitis, which resolved immediately after treatment. The reported incidence of grade 3 or higher acute toxicity after RT for MSCs ranged widely from 19 to 75% [5.8]. Regarding late toxicity, grade 2 toxicity, such as dysphagia and dysgeusia, were observed in 2 patients (11%), and there was no grade 3 or higher toxicity. There are few reports separately describing the rates of acute and late toxicities. Ebara *et al*. reported that grade 2 or higher late toxicity was observed in 11% of patients who underwent selective arterial chemo-infusion for MSCs [8], which appears to be consistent with our results. We consider that the toxicity profile is acceptable, even though the irradiated field tends to be larger in treating MSCs with neck lymph node metastasis compared with MSCs without neck lymph node metastasis.

This study had some limitations. First, it was designed as a single institutional retrospective study with a small number of patients. Second, there was heterogeneity in the RT methods used, such as 3D-CRT and IMRT, and in the dose. Third, diagnosis of lymph node metastasis had some uncertainty. Most of the patients were clinically diagnosed by image findings. However, at the same time, most of the clinically diagnosed patients showed multiple swollen lymph node with high abnormal uptake value of FDG.

Although there were some limitations, we still expect that this study will provide beneficial information to clinicians because of a lack of data regarding treatment results of definitive RT with concurrent chemotherapy for MSCs with neck lymph node metastasis. Further study with a larger number of patients will be necessary to elucidate the optimal treatment method in MSC patients with neck lymph node metastasis. In conclusion, we described the results of definitive RT with concurrent chemotherapy for MSC patients with neck lymph node metastasis. The 2-year LC, PFS and OS rates for all patients were 34, 31 and 46%, respectively. Local recurrence was a frequent pattern of failure. Longer follow-up with a larger number of patients will be necessary to clarify whether sufficient radiation dose coverage of the tumor with IMRT could achieve a better LC rate or not.

## CONFLICT OF INTEREST

None declared.

## FUNDING

The authors have no funding source to be declared.
